# Health-promoting factors among students in higher education within health care and social work: a cross-sectional analysis of baseline data in a multicentre longitudinal study

**DOI:** 10.1186/s12889-022-13690-z

**Published:** 2022-07-09

**Authors:** Inger Ahlstrand, Ingrid Larsson, Margaretha Larsson, Aimée Ekman, Lena Hedén, Katja Laakso, Ulrika Lindmark, Håkan Nunstedt, Lena Oxelmark, Sandra Pennbrant, Annelie J. Sundler, Jenny Hallgren

**Affiliations:** 1 Department of Rehabilitation, School of Health and Welfare, Jönköping University, Jönköping, Sweden; 2 School of Health and Welfare, Halmstad University, Box 823, S-30118 Halmstad, Sweden; 3 School of Health and Education, University of Skövde, Skövde, Sweden; 4 Department of Social Work, School of Health and Welfare, Jönköping University, Jönköping, Sweden; 5 Faculty of Caring Science, Work Life and Social Welfare, University of Borås, Borås, Sweden; 6 Institute of Health and Care Sciences, The Sahlgrenska Academy, University of Gothenburg, Gothenburg, Sweden; 7 Department of Health Sciences, Karlstad University, Karlstad, Sweden; 8 Centre for Oral Health, School of Health and Welfare, Jönköping University, Jönköping, Sweden; 9 Department of Health Sciences, University West, Trollhättan, Sweden

**Keywords:** Health and health-promoting resources, Health behavior, Healthy lifestyles, Higher education, Occupational Balance Questionnaire, Salutogenesis, Salutogenic Health Indicator Scale, Sense of coherence, Students’ health

## Abstract

**Background:**

Educational environments are considered important in strengthening students’ health status and knowledge, which are associated with good educational outcomes. It has been suggested to establish healthy universities based on a salutogenic approach – namely, health promotion. The aim of this study was to describe health-promoting resources and factors among first-semester students in higher education in healthcare and social work.

**Methods:**

This cross-sectional study is based on a survey distributed among all students in seven healthcare and social work programmes at six universities in southern Sweden. The survey was carried out in 2018 using a self-reported, web-based questionnaire focussing on general health and well-being, lifestyle factors together with three validated instruments measuring health-promoting factors and processes: the Sense of Coherence (SOC) scale, Salutogenic Health Indicator Scale (SHIS) and Occupational Balance Questionnaire (OBQ).

**Results:**

Of 2283 students, 851 (37.3%) completed the survey, of whom 742 (87.1%) were women; 722 (84.8%) were enrolled on healthcare programmes, and 129 (15.2%) were enrolled on social work programmes. Most reported good general health and well-being (88.1% and 83.7%, respectively). The total mean scores for the SOC scale, SHIS and OBQ were, respectively, 59.09 (*SD* = 11.78), 44.04 (*SD* = 9.38) and 26.40 (*SD* = 7.07). Well-being and several healthy lifestyles were related to better general health and higher SOC, SHIS and OBQ scores. Multiple linear and logistic regressions showed that perceived well-being and no sleeping problems significantly predicted higher general health and higher SOC, SHIS and OBQ scores. Being less sedentary and non-smoking habits were significant predictors of higher SOC.

**Conclusions:**

Swedish students in higher education within the healthcare and social work sector report good general health and well-being in the first semester, as well as health-promoting resources (i.e. SOC, SHIS and OBQ), and in some aspects, a healthy lifestyle. High-intensity exercise, no sleeping problems and non-smoking seem to be of importance to both general health and health-promotive resources. This study contributes to knowledge about the health promotive characteristics of students in the healthcare and social work fields, which is of importance for planning universities with a salutogenic approach.

## Background

Health-promoting factors are of great importance during education and in preparing for future work and professional roles [1]. A healthy work-life is considered important, and health promotion is required for health service providers to ensure a sustainable working life. Nevertheless, more knowledge is needed about the relationship between education and health and factors that may be important for future working-life balance among individuals [2].

Health promotion is described as comprising processes that can support people to manage and improve their health [3], with the result that they choose healthy behaviours. Globally, related research has largely focused on students’ health behaviours, including in Southern Europe [4], Asia [5], Australia [6] and the United States [7]. Published studies have shown that factors that including household income [8] and lifestyle factors, such as dietary patterns [9], physical activity, strength training [10] and sleep [11], can be associated with students’ health and academic performance. Other studies have illustrated that students enrolled on higher education programmes can suffer from various physical problems, such as neck, shoulder and/or back pain [12], and daytime sleepiness and sleep debt, which can put them at risk for mental health disorders, such as depression and anxiety [13]. Based on this, health-promoting strategies, such as physical activity, are recommended by the World Health Organization [14]. Another positive association that promotes health has been found between enrolment on health-related university courses and students’ health promotion competencies [15, 16]. Thus, strengthening students’ health literacy can be a way to support their health-promoting resources. A study that included an intervention using didactical approaches (e.g. peer mentoring) and team teaching among students enrolled on bachelor-level health and social work programmes reported that students in the intervention group showed significant effects on self- and social competencies compared with controls [17].

This study is grounded in the theoretical framework of salutogenesis [18], which is the theory behind health-promotion that aiming to promote health as opposed to targeting factors that cause disease [19]. Essential to salutogenic theory is an understanding of health as a process that exists along a continuum namely, the health/disease continuum. The most important concept in the theory is the Sense of Coherence (SOC) with the three dimensions, comprehensibility, meaningfulness and manageability. These dimensions represent a combination of people’s ability to assess and understand the situation they are in and to find meaning, move in a health-promotional direction and manage the situation [19, 20]. A study has shown a strong relationship between SOC and health and well-being [19]. Influenced by salutogenic theory, health has also been described by Bringsén et al. (2009, p. 4) as a positive subjective experience of oneself as a whole, which is why a holistic description of health, including an individuals’ cognitive, physical and psychosomatic health, which is important to consider in health promotion research [21]. Among students in higher education, SOC has only been studied sparsely in relation to health promotion factors; however, studies on this topic from Asia have indicated that a lower level of perceived stress, and higher levels of healthy behaviours and integration at university, are related to a strong SOC [22, 23]. Balance in life is an important health-promoting factor [24]. Research has shown that occupational balance has an impact on health professionals [25] and could also be an important promotive factor for students within the healthcare and social work fields.

Recently, it has been emphasised that there is a need to focus on health promotion approaches to strengthen positive aspects [19], not least for students on higher education programmes [26, 27]. The integration of a health promotion approach in health education curricula is important [28]. It is necessary to identify health-promoting factors [29] and to find appropriate interventions among students during their education [30]. Therefore, improving students’ health and health status are important to achieving good educational outcomes [31]. However, more knowledge is needed to understand how a salutogenic approach can create good conditions for developing students’ abilities and resources to feel well during their studies, thus making them better prepared for future sustainable working life. Knowledge of the working environment from a salutogenic perspective is not fully understood in healthcare and social work programmes, which is why there is a need for studies to address this gap. Knowledge and awareness of health-promoting behaviour while at university can improve students’ ability to deal with stress and negative load in connection with professional preparation. This is important knowledge that can form the basis for understanding the transition into and the establishment of the professional role. Therefore, this study presents baseline data from a longitudinal multicentre study focusing on health-promoting resources and factors for sustainable studies on higher education programmes and, by extension, a sustainable working life. The aim was to describe health, health-promoting resources and lifestyle factors as reported by first-year bachelor students in healthcare and social work programmes. In addition, we studied how health, health-promoting resources and lifestyle factors were associated.

## Methods and design

This is a cross-sectional study using baseline data from a multicentre longitudinal study involving Swedish students enrolled on higher education programmes in the healthcare and social work fields [32]. Public health issues are addressed by both healthcare and social work, albeit in different ways. Whereas the former focuses more directly on individuals and their health status, the latter deals primarily with social change and development for individuals, families, and groups, with the aim of achieving enhanced well-being and health [33]. The Strengthening the Reporting of Observational Studies in Epidemiology (STROBE) guidelines have been followed to strengthen the trustworthiness of the study [34].

### Setting and participants

The study was conducted within the Swedish framework for ‘Health Research in Collaboration’, involving six universities in southern Sweden. The qualifications sought in the included higher educational programmes were; biomedical laboratory scientist, diagnostic radiology nurse, occupational therapist, physiotherapist, registered dental hygienist, registered nurse and social worker. All students, who started their higher education in one of these programmes at the six universities in 2018, were eligible and invited to participate in the study. The only exclusion criterion was students not speaking/reading Swedish.

### Data collection

Data were collected via a survey distributed to all eligible students in the selected programmes and universities during either the spring and autumn of 2018. The survey was performed using a self-reported, web-based questionnaire (esMaker NX3 software), including questions regarding sociodemographic characteristics and questionnaires covering salutogenic factors, the individual’s health, personal resources and health behaviour. The survey was sent out in the middle of the first semester, and three reminders were sent after that point [32].

### Measurements

At baseline, a web-based questionnaire was used that included questions related to background that is, the students’ characteristics. It included questions about demographic characteristics, reason for choosing the education programme, general health and well-being; and health-promoting resources as measured by the SOC scale [18], the Salutogenic Health Indicator Scale (SHIS) [21] and the Occupational Balance Questionnaire (OBQ) [35]. Questions related to healthy lifestyles were based on the Swedish Public Health Survey [36].

#### Background data

Questions covering demographics were used to elicit information on participants’ sex, age, ethnicity, family situation and residential area. Reasons for choosing the education programme (13 questions) were used to further describe the students’ characteristics to understand their intentions and motivations in applying for the specific higher education programme and establishing themselves in the profession.

#### Health and health-promoting resources

*General health* and *perceived well-being* were measured as one overall single question and dichotomised as excellent/very good/good (1) or less good/bad (0).

*The Sense of Coherence* (SOC) was measured using the 13-item SOC scale [18], examining the individual’s health-promoting resources. The SOC scale’s items relate to comprehensibility (five items), manageability (four items) and meaningfulness (four items), and a 7-point semantic scale is used to respond to each item. The total score ranges from 13 to 91, and a high score indicates a strong SOC. The scale has been translated into Swedish [37]. It has good psychometric properties and high validity and reliability, and it has been found to be a good indicator of health [38].

The questionnaire included an abbreviated version of the SHIS (12-item) [21]. SHIS is associated with salutogenic and holistic descriptions of health, including cognitive, physical and psychosomatic health. This scale examines intrapersonal characteristics and interactive functions on a 6-point scale, ranging from negative (scored at 1) to positive (scored at 6). The missing item on the SHIS scale was about ‘having energy’. This item was not included in the questionnaire by mistake. The total score ranges from 11 to 66, and a high score indicates better health. The SHIS’s validity has been shown to be high [21].

The *OBQ* [35] measures occupational balance and focuses on satisfaction with the amount and variation of occupations. It comprises 11 items with a 4-point ordinal scale ranging from ‘completely disagree’ (scored at 0) to ‘completely agree’ (scored at 3). High scores reflect high levels of experienced occupational balance. The OBQ was developed in Sweden and has good content validity, good internal consistency and sufficient test–retest reliability [24, 35].

#### Healthy lifestyles

The Swedish Public Health Survey [36] is a national survey conducted every other year that aims to investigate public health. It includes questions related to physical and mental health, drug consumption and lifestyle, among other topics. Eight questions were drawn from the Swedish Public Health Survey that assess healthy lifestyles; high-intensity exercise (dichotomised as yes > 60–90 min/week (1), no < 30–60 min/week (0)), moderate-intensity physical activity i.e. everyday physical activities, (dichotomised as yes > 150 min/week (1), no < 150 min/week (0)), sedentary (more than 10 h (1), no (0)), sleeping problems (yes (1), no (0)), daily intake of vegetables (yes (1), less frequency than daily intake (0)), consumption of alcohol (yes (1), < once per month/no (0)), smoking (yes (1), seldom/no (0)) and daily snuff (oral tobacco) (yes (1), seldom/no (0)).

#### Outcome variables

The outcome variables in this study were general health, SOC, SHIS and OBQ.

### Data analysis

Descriptive analysis was used to describe the background characteristics and reasons for choosing the selected programme. The χ^2^ test, *t*-test, Mann–Whitney U test and Kruskal–Wallis test were used to describe health-promoting resources and differences in general health, healthy lifestyles (perceived well-being, physical exercise, everyday physical activities, being sedentary more than 10 h per day, sleeping problems, intake of vegetables, consumption of alcohol, smoking, daily snuff intake) in terms of general health, SOC, SHIS and OBQ scores, respectively. The Shapiro–Wilk statistic was used to test normality. If the distributions were not normal or ordinal, the Mann–Whitney U-test and Kruskal–Wallis test were used. Factors that were significantly associated with general health, SOC, SHIS and OBQ from the χ^2^ test, *t*-test and Mann–Whitney U test, were included in a multiple logistic regression (general health) and a multiple linear regression analysis (SOC, SHIS and OBQ). A *p*-value < 0.05 was considered significant. All data were analysed using SPSS Statistics 27.

## Results

### Students’ characteristics

A total of 2283 students were invited to participate in the study, of whom 851 (37%) completed the baseline survey. The participants were all students in programmes in healthcare and social work fields, studying for a qualification as one of the following professions: biomedical laboratory scientists (*n* = 51), diagnostic radiology nurses (*n* = 26), occupational therapists (*n* = 58), physiotherapists (*n* = 24), registered dental hygienists (*n* = 27), registered nurses (*n* = 536) and social workers (*n* = 129).

Among the 851 included students in higher education programmes in the healthcare and social work sectors, 742 (87.1%) were women and 106 (12.9%) were men. The respondents had a mean age of 28 and a range of 21–61 years. A total of 137 (16.2%) of the students were foreign-born, and 255 (30.0%) reported that one or both of their parents were foreign-born. Living in a rural area was reported by 109 (12.7%) of respondents, and 148 (17.4%) of the students were living alone (Table 1).

**Table 1:** Participants’ characteristics and health-promoting lifestyle factors, totally, in sex and educational program

	Totally *N* = 851	Women *n* = 742	Men *n* = 106	Biomedical laboratory scientist, (Clin Phys) *n* = 18	Biomedical laboratory scientist, (Lab Med) *n* = 33	Diagnostic radiology nurses *n* = 26	Occupational therapist *n* = 58	Physio-therapist *n* = 24	Registered dental hygienists *n* = 27	Registered nurse *n* = 536	Social worker *n* = 129
Age, years, Md (IQR)	26 (8)	26 (7)	28 (8)	23.5 (8)	25 (5)	25.5 (11)	27 (8)	26 (5.75)	26 (8)	26.5 (9)	25 (6)
Gender, women, n (%)	742 (87.1)			16 (88.9)	29 (87.9)	23 (88.5)	49 (84.5)	17 (70.8)	24 (88.9)	473 (88.2)	110 (85.3)
Born in Sweden (Yes) n (%)	714 (83.8)	624 (84.1)	87 (82.1)	13 (72.2)	28 (84.8)	22 (84.6)	49 (84.5)	22 (91.7)	18 (66.7)	445 (83.0)	116 (89.9)
Both parents born in Sweden n (%)	596 (70.0)	526 (70.9)	67 (63.2)	8 (44.4)	22 (66.7)	15 (57.7)	38 (65.5)	22 (91.7)	9 (33.3)	376 (70.1)	105 (81.4)
One parent born in Sweden n (%)	88 (10.3)	74 (10.0)	14 (13.2)	2 (11.1)	4 (12.1)	3 (11.5)	8 (13.8)	1 (4.2)	2 (7.4)	57 (10.6)	11 (8.5)
Both parents born in another country n (%)	168 (19.7)	145 (19.5)	23 (21.7)	7 (38.9)	7 (21.2)	6 (23.1)	10 (17.2)	1 (4.2)	14 (51.9)	108 (20.1)	47 (36.4)
Residential area (rural) n (%)	109 (12.7)	100 (13.5)	8 (7.5)	2 (11.1)	2 (6.1)	2 (7.7)	5 (8.6)	1 (4.2)	4 (14.8)	76 (14.2)	15 (11.6)
*Who do you share a home with?*											
Living alone	148 (17.4)	123 (16.6)	25 (23.6)	5 (27.8)	3 (9.1)	7 (26.2)	10 (17.2)	7 (29.2)	7 (25.9)	84 (15.7)	25 (19.4)
Parents /siblings	202 (23.7)	174 (23.5)	26 (24.5)	6 (33.3)	10 (30.3)	6 (23.1)	15 (25.9)	7 (29.2)	7 (25.9)	125 (23.3)	26 (20.2)
Husband/wife/ partner	355 (41.7)	317 (42.7)	36 (34.0)	3 (16.7)	13 (39.4)	9 (34.6)	21(36.2)	8 (33.3)	8 (29.6)	236 (44.0)	56 (43.4)
Other adults	73 (8.6)	60 (8.1)	13 (12.3)	2(11.1)	5 (15.2)	1 (3.8)	8 (13.8)	3 (12.5)	1 (3.7)	37 (6.9)	16 (12.4)
Children	97 (11.4)	89 (12.0)	8 (7.5)	2 (11.1)	3 (9.1)	3 (11.5)	5 (8.6)	0	4 (14.8)	71 (13.2)	9 (7.0)
*Health-promoting lifestyle factors*											
General health (Good) n (%)	751 (88.1)	660 (88.9)	90 (84.9)	17 (94.4)	29 (87.9)	21 (80.8)	54 (93.1)	23 (95.8)	24 (88.9))	472 (88.1)	110 (85.3)
Perceived wellbeing (Good) n (%)	713 (83.7)	620 (83.6)	92 (86.8)	16 (88.9)	29 (87.9)	21 (80.8)	54 (93.1)	19 (79.2)	24 (88.9)	439 (81.9)	110 (85.3)
Smoking n (%)	125 (14.7)	111 (15.0)	14 (13.2)	2 (11.1)	5 (15.2)	1 (3.8)	10 (17.2)	2 (8.3)	3 (11.1)	86 (16.0)	16 (12.4)
Daily snuff n (%)	113 (13.3)	83 (11.2)	30 (28.3)	2 (11.1)	3 (9.1)	3 (11.5)	8 (13.8)	1 (4.2)	0	76 (14.2)	20 (15.5)
Consumption of alcohol (Never/ones a month) n (%)	491 (57.6)	434 (58.5)	53 (50.0)	9 (50.0)	18 (54.5)	14 (53.8)	32 (55.2)	14 (58.3)	22 (81.5)	324 (60.4)	58 (45.0)
Daily intake of vegetables n (%)	584 (60.0)	471 (63.5)	62 (58.5)	11 (61.1)	23 (69.7)	16 (61.5)	42 (70.7)	22 (91.7)	14 (51.9)	323 (60.3)	83 (64.3)
Physical exercise n (%)	423 (47.5)	358 (48.2)	64 (60.4)	7 (38.9)	18 (54.5)	12 (46.2)	35 (60.3)	23 (95.8)	10 (37.0)	262 (48.9)	55 (42.6)
Everyday physical activities n (%)	291 (32.7)	256 (34.5)	34 (32.1)	4 (22.2)	8 (24.2)	8 (30.8)	21 (36.2)	18 (75.0)	7 (25.9)	189 (35.3)	36 (27.9)
Sedentary more than 10 h n (%)	230 (25.8)	196 (26.4)	32 (30.2)	7 (38.9)	10 (30.3)	9 (34.6)	18 (31.0)	5 (20.8)	10 (37.0)	123 (22.9)	48 (37.2)
Sleeping problems n (%)	343 (40.3)	300 (40.4)	39 (36.8)	12 (66.7)	13 (39.4)	12 (46.2)	19 (32.8)	11 (45.8)	9 (33.3)	223 (41.6)	44 (34.1)

### Reason for choosing the programme

The most common reasons (87.0%–92.8%) for choosing the selected programme and career were employment security, working with people, helping people, a broad education, opportunities to follow various career paths, varying tasks, intellectually stimulating, getting a permanent job and use of knowledge from the programme. Teamwork and closeness to a university were the least common reasons (61.8%–64.0%). There were some differences between the reasons for choosing the programme (Table 2).

**Table 2:** Reasons for choosing the selected program and career

	Total *N* = 851	Women *n* = 742	Men *n* = 106	Biomedical laboratory scientist, (Clin Phys) *n* = 18	Biomedical laboratory scientist, (Lab Med) *n* = 33	Diagnostic radiology nurses *n* = 26	Occupational therapist *n* = 58	Physio-therapist *n* = 24	Registered dental hygienists *n* = 27	Registered nurse *n* = 536	Social worker *n* = 129
Teamwork, n (%)	527 (61.8)	463 (62.4)	62 (58.5)	8 (44.5)	11 (33.3)	19 (73.0)	33 (56.9)	12 (50.0)	10 (37.0)	370 (69.1)	63 (48.8)
Employment security, n (%)	791 (92.8)	693 (93.4)	94 (88.7)	18 (100)	30 (90.9)	25 (86.2)	53 (91.4)	23 (95.8)	23 (85.1)	503 (93.8)	115 (89.2)
Working with people, n (%)	778 (91.4)	682 (91.9)	93 (87.7)	16 (88.9)	15 (45.5)	22 (84.6)	55 (94.8)	22 (91.7)	21 (77.7)	506 (94.4)	120 (93.1)
Helping people, n (%)	791 (92.8)	705 (95.0)	99 (93.4)	18 (100)	30 (90.9)	25 (96.2)	56 (96.6)	24 (100)	22 (81.4)	510 (95.1)	122 (94.6)
The education is broad, n (%)	750 (88.0)	657 (88.5)	90 (84.9)	17 (94.4)	31 (94.0)	23 (88.5)	54 (93.1)	23 (95.9)	22 (81.5)	482 (90.0)	123 (95.3)
Be able to choose different career paths, n (%)	759 (89.1)	662 (89.2)	93 (87.8)	13 (72.2)	29 (87.9)	15 (57.7)	53 (91.4)	21 (87.5)	16 (59.2)	490 (91.4)	121 (93.8)
Varying tasks, n (%)	744 (87.3)	653 (88.0)	87 (82.1)	17 (94.4)	30 (90.9)	24 (92.4)	50 (86.2)	22 (91.7)	18 (66.6)	476 (88.8)	106 (82.1)
The work is intellectual stimulating, n (%)	725 (85.1)	639 (86.1)	82 (77.4)	17 (94.4)	30 (90.9)	24 (92.4)	50 (86.2)	22 (91.7)	18 (66.6)	458 (85.4)	105 (81.4)
Get a permanent job, n (%)	741 (87.0)	655 (88.2)	82 (77.3)	16 (88.9)	29 (87.9)	25 (96.1)	52 (89.6)	21 (87.5)	23 (75.2)	465 (86.8)	109 (84.5)
Closeness to University, n (%)	545 (64.0)	475 (64.1)	68 (64.2)	7 (38.9)	22 (63.6)	17 (65.4)	38 (65.5)	15 (62.5)	15 (55.5)	348 (55.1)	82 (63.6)
Use the knowledge from my education, n (%)	778 (91.3)	680 (91.6)	95 (89.6)	18 (100)	30 (90.9)	26 (100)	52 (89.7)	24 (100)	23 (85.1)	490 (91.5)	114 (88.3)

### Health and health-promoting resources

Overall, 751 (88.1%) of the students reported good general health and 713 (83.7%) reported good general well-being (Table 1). The mean total SOC score was 59.09 (*SD* = 11.78), and the means for the subscales were as follows: Comprehensibility, 20.69 (*SD* = 5.39); Meaningfulness, 20.62 (*SD* = 4.10); and Manageability, 17.82 (*SD* = 4.37). The total mean SHIS score was 44.04 (*SD* = 9.38). There were significant differences between women and men in all health-promoting resources reported, besides concerning OBQ and the total SOC score. Categorised into the three SOC dimensions, men had statistically significantly higher Comprehensibility (95% confidence interval [CI]: 0.37, 2.57; *p* = 0.09) and Manageability scores (95% CI: 0.51, 2.30; *p* = 0.02) than women did, but women reported higher Meaningfulness scores (95% CI: − 1.76, − 0.10; *p* = 0.03). There were no statistically significant differences between students in different educational programmes for the OBQ or the total SOC score. However, there were significant differences in regard to the SHIS, where occupational therapy students had a statistically significantly higher SHIS score (60.8; *SD* = 11.0) compared with all other student groups. Table 3 shows more details and health-promoting resources for each educational programme.

**Table 3:** Health-promoting resources measured using SOC and subscales, SHIS and OBQ

	Total *N* = 851	Women *n* = 742	Men *n* = 106	*p-value*	Cohens-d	Biomedical laboratory scientist, (clin phys) *n* = 18	Biomedical laboratory scientist, (lab med) *n* = 33	Diagnostic radiology nurses *n* = 26	Occupational Therapist *n* = 58	Physio-therapist *n* = 24	Registered dental hygienists *n* = 27	Registered nurse *n* = 536	Social worker *n* = 129
SOC Total 13–91 (sd)	59.09 (11.78)	58.87 (11.84)	61.11 (10.86)	.083	.191	57.39 (10.94)	56.45 (13.23)	59.73 (12.98)	60.82 (11.00)	57.00 (12.63)	56.73 (11.53)	59.40 (11.78)	58.67 (11.52)
Comprehensibility 1–35 (sd)	20.69 (5.39)	20.52 (5.45)	21.99 (4.75)	**.011**	.273	21.06 (4.95)	18.76 (6.09)	21.12 (5.75)	21.20 (5.47)	19.54 (5.32)	19.08 (5.83)	20.93 (5.38)	20.43 (5.03)
Meaningfulness 1–28 (sd)	20.62 (4.10)	20.76 (4.01)	19.83 (4.49)	**.030**	.228	19.39 (4.70)	19.79 (4.33)	20.62 (4.72)	21.39 (3.47)	20.00 (4.10)	19.92 (3.38)	20.67 (4.11)	20.58 (4.07)
Manageability 1–28 (sd)	17.82 (4.37)	17.66 (4.38)	19.07 (4.13)	**.002**	.324	16.94 (2.88)	17.91 (4.67)	18.00 (4.00)	18.23 (4.07)	17.46 (4.62)	17.73 (4.38)	17.79 (4.35)	17.65 (4.67)
SHIS 11–66 (sd)	44.04 (9.38)	43.83 (9.37)	45.86 (9.10)	.**032**	.217	42.83 (8.56)	46.09 (8.19)	43.65 (9.25)	47.40 (8.07)	42.33 (8.99)	39.81 (9.98)	43.71 (9.77)	44.81 (8.19)
OBQ 11–44 (sd)	26,40 (7.07)	26.28 (7.11)	27.28 (6.80)	**.**137	.141	29.38 (6.42)	27.77 (6.21)	26.80 (6.27)	26.91 (7.35)	26.57 (6.78)	24.68 (6.78)	26.12 (7.27)	26.83 (6.62)

*SOC* Sense Of Coherence, *SHIS* Salutogenic Health Indicator Scale, *OBQ* Occupational Balance Questionnaire

### Healthy lifestyles

In the total group, 726 (85.3%) of respondents were non-smokers and 738 (86.7%) were not daily snuff users; 491 (57.6%) reported low alcohol consumption (never or once a month) consumption and 584 (60.0%) had a daily intake of vegetables. High-intensity exercise for more than 60 min/week and moderate-intensity physical activity for more than 150 min/week were performed by 423 (47.5%) and 291 (32.7%) of respondents, respectively, whereas 230 (25.8%) were sedentary for more than 10 h per day. Moreover, 508 (59.7%) reported no sleeping problems. The students’ healthy lifestyles in total and in each educational programme are presented in Table 1.

### Associations between health-promoting resources and lifestyle factors

The participants reporting better general health had better perceived well-being, (*p* < 0.001), performed high-intensity exercise (*p* < 0.001), were less sedentary (*p* = 0.031), had no sleeping problems (*p* < 0.001), consumed vegetables daily (*p* < 0.001), had low or no consumption of alcohol (*p* = 0.009) and were non-smokers (*p* = 0.005). Significantly higher SOC, SHIS and OBQ were seen among participants who had better general health (*p* < 0.001, *p* < 0.001, *p* < 0.001), perceived well-being (*p* < 0.001, *p* < 0.001, *p* < 0.001), no sleeping problems (*p* < 0.001, *p* < 0.001, *p* < 0.001), and low or no consumption of alcohol (*p* = 0.032, *p* < 0.001, *p* = 0.002). Being less sedentary and having a daily intake of vegetables were associated with higher SOC (*p* < 0.001), *p* < 0.001) and SHIS (*p* < 0.001, *p* = 0.003). Regarding exercise and physical activity, high-intensity exercise was associated with higher SHIS (*p* = 0.003) and OBQ (*p* < 0.001), whereas moderate-intensity physical activity was associated with higher SOC. However, the use of daily snuff was not significantly associated with general health (*p* = 0.164) or higher SOC (*p* = 0.429), SHIS (*p* = 0.577) or OBQ (*p* = 0.206) (Table 4).

**Table 4:** Differences in general health, perceived wellbeing and healthy lifestyles on general health, SOC, SHIS, and OBQ respectively

*General health*^*b*^	Yes, n (%)	No, n (%)	*p-*value
Sex (Woman)	660 (89.6)	104 (86.6)	0.354
Perceived wellbeing (Good)	672 (94.5)	77 (58.3)	< 0.001
High-intensity exercise (Physical exercises) > 60–90 min/week	398 (53.1)	23 (24.5)	< 0.001
Moderate-intensity physical activity (Everyday physical activities) > 150 min/week	260 (34.7)	29 (30.9)	0.462
Sedentary > 10 h/day	193 (85.0)	558 (90.3)	0.031
Sleeping problems	279 (82.1)	468 (93.4)	< 0.001
Daily intake of vegetables	487 (64.8)	45 (47.9)	< 0.001
Consumption of alcohol	421 (86.4)	330 (92.2)	0.009
Smoking	102 (81.6)	648 (90.1)	0.005
Daily snuff	96 (85.0)	649 (89.4)	0.164
*SOC*^*a*^	Yes, Mean (sd)	No, Mean (sd)	*p-*value
Sex (Woman)	58.87 (11.84)	61.11 (10.86)	0.071
General health (Good)	60.20 (11.44)	50.61 (11.13)	< 0.001
Perceived wellbeing (Good)	61.31 (10.83)	47.02 (9.12)	< 0.001
High-intensity exercise (Physical exercises) > 60–90 min/week	59.89 (11.99)	58.31 (11.53)	0.053
Moderate-intensity physical activity (Everyday physical activities) > 150 min/week	60.87 (11.99)	58.18 (11.58)	0.002
Sedentary > 10 h/day	56.53 (11.37)	60.04 (11.79)	< 0.001
Sleeping problems	54.21 (11.19)	62.34 (11.03)	< 0.001
Daily intake of vegetables	60.30 (11.90)	57.05 (11.29)	< 0.001
Consumption of alcohol	58.34 (11.53)	60.11 (12.04)	0.032
Smoking	56.30 (11.82)	59.63 (11.70)	0.004
Daily snuff	58.21 (11.10)	59.16 (11.87)	0.429
*SHIS*^*a*^	Yes, Mean (sd)	No, Mean (sd)	*p*-value
Sex (Woman)	43.83 (9.37)	45.86 (9.10)	0.037
General health (Good)	44.95 (8.91)	36.54 (9.73)	< 0.001
Perceived wellbeing (Good)	45.71 (8.56)	34.91 (8.35)	< 0.001
High-intensity exercise (Physical exercises) > 60–90 min/week	44.99 (9.13)	43.09 (9.51)	0.003
Moderate-intensity physical activity (Everyday physical activities) > 150 min/week	44.82 (9.20)	43.63 (9.44)	0.078
Sedentary > 10 h/day	42.16 (10.13)	44.73 (9.00)	< 0.001
Sleeping problems	39.81 (8.84)	46.88 (8.67)	< 0.001
Daily intake of vegetables	44.37 (9.44)	43.44 (9.25)	0.003
Consumption of alcohol	42.99 (9.35)	45.47 (9.25)	< 0.001
Smoking	42.64 (9.82)	44.26 (9.29)	0.075
Daily snuff	43.54 (8.79)	44.07 (9.48)	0.577
*OBQ*^*c*^	Yes, Mean (sd)	No, Mean (sd)	*p*-value
Sex (Woman)	26.28 (7.11)	27.28 (6.80)	0.137
General health (Good)	26.84 (7.02)	22.73 (6.40)	< 0.001
Perceived wellbeing (Good)	27.24 (6.82)	21.87 (6.66)	< 0.001
High-intensity exercise (Physical exercises) > 60–90 min/week	27.19 (7.18)	26.11 (6.85)	< 0.001
Moderate-intensity physical activity (Everyday physical activities) > 150 min/week	26.42 (7.12)	26.39 (7.01)	0.697
Sedentary > 10 h/day	26.60 (7.25)	26.32 (7.01)	0.842
Sleeping problems	24.74 (6.78)	27.50 (7.05)	< 0.001
Daily intake of vegetables	26.54 (7.18)	26.11 (6.85)	0.357
Consumption of alcohol	25.75 (6.97)	27.29 (7.12)	0.002
Smoking	26.09 (6.82)	26.43 (7.11)	0.525
Daily snuff	25.42 (7.01)	26.51 (7.01)	0.206

^a^ T-test, ^b^ Chi-square, ^c^ Mann–Whitney

When significantly associated factors were entered, based on the univariate analyses in multiple linear regressions, perceived well-being and no sleeping problems were significant predictors of better general health and higher SOC, SHIS and OBQ scores. In addition, performing high-intensity exercise predicted better general health; in turn, better general health predicted higher SHIS scores. Being less sedentary and non-smokers were significant predictors of higher SOC (Tables 5,6,7,8).

**Table 5:** Multiple logistic regression of the predictive factors of general health

Variables	Dependent variable General Health			
	B (SE)	Odds ratio	95% CI	*p*-value
Perceived wellbeing	2.239 (0.261)	9.387	5.626– 15.661	< 0.001
High-intensity exercise (Physical exercises) > 60–90 min/week	1.138 (0.278)	3.120	1.810– 5.378	< 0.001
Sedentary > 10 h/day	-0.115 (0.278)	0.891	0.527 – 1.506	0.668
Sleeping problems	-0.526 (0.263)	0.591	0.353 – 0.989	0.045
Daily intake of vegetables	0.402 (0.252)	1.495	0.912 – 2.452	0.111
Consumption of alcohol	-0.478 (0.279)	0.613	0.933 – 2.788	0.087
Smoking	-0.736 (0.315)	0.479	0.258 – 0.889	0.020

*CI* Confidence interval, *R*^*2*^ = 0.158 (Cox & Snell), σ 0.313 (Nagelkerke), Model χ2 = 143.873, *p* =  < 0.001

**Table 6:** Multiple linear regression analysis of the predictive factors of SOC

Variables	Dependent variable SOC			
	β	Standard error (β)	95% CI	*p*-value
General health	1.993	1.237	-0.435 – 4.420	0.101
Perceived wellbeing	11.118	1.093	8.971 – 13.264	< 0.001
Moderate-intensity physical activity (Everyday physical activities) > 150 min/week	2.033	0.757	0.547 – 3.519	0.007
Sedentary > 10 h/day	-1.760	0.811	0.168 – 3.352	0.030
Sleeping problems	-5.565	0.751	-7.039 – -4.092	< 0.001
Daily intake of vegetables	1.217	0.749	-0.253 – 2.686	0.105
Consumption of alcohol	-0.407	0.733	-1.845 – 1.032	0.579
Smoking	-2.122	0.999	-4.083 – -0.164	0.034

*CI* Confidence interval, *R*^*2*^ = 0.275; ANOVA: F = 38.25, *p* =  < .001

**Table 7:** Multiple linear regression analysis of the predictive factors of SHIS

Variables	Dependent variable SHIS			
	β	Standard error (β)	95% CI	*p*-value
Sex	-1.444	0.853-	-3.119 – 0.231	0.091
General health	2.830	0.994	0.878 – 4.782	0.005
Perceived wellbeing	7.737	0.861	6.047 – 9.427	< 0.001
High-intensity exercise (Physical exercises) > 60–90 min/week	0.486	0.588	-0.669 – 1.641	0.669
Moderate-intensity physical activity (Everyday physical activities) > 150 min/week	0.880	0.607	-0.311 – 2.071	0.147
Sedentary > 10 h/day	-1.250	0.642	-2.510 – 0.010	0.052
Sleeping problems	-5.160	0.589	-6.316 – -4.004	< 0.001
Daily intake of vegetables	0.285	0.590	-1.445 – 0.874	0.629
Consumption of alcohol	-1.163	0.572	-2.286 – -0.050	0.042

*CI* Confidence interval, *R*^*2*^ = 0.273; ANOVA: F = 34.38, *p* =  < .001

**Table 8:** Multiple linear regression analysis of the predictive factors of OBQ

Variables	Dependent variable OBQ			
	β	Standard error (β)	95% CI	*p*-value
General health	1.173	0.859	-0.514 – 2.859	0.173
Perceived wellbeing	4.150	0.738	2.701 – 5.598	< 0.001
High-intensity exercise (Physical exercises) > 60–90 min/week	1.026	0.487	0.069 – 1.983	0.036
Sleeping problems	-1.715	0.506	-2.707 – -0.723	0.001
Consumption of alcohol	-0.749	0.492	-0.217 – 1.714	0.217

*CI* Confidence interval, *R*^*2*^ = 0.106; ANOVA: F = 18.51, *p* =  < .001

## Discussion

This study describes the associations between general health, perceived well-being, health-promoting resources measured by the SOC, SHIS and OBQ scores and healthy lifestyles, comparing results from students in several higher education programmes. The main results showed that of the first-year students in higher education within health care and social work, most seemed to report good general health and well-being, and possessed health-promoting resources and a health-promoting lifestyle. Better general health was also associated with better perceived well-being, high-intensity exercise, no sleeping problems and not smoking. Associations related to health-promoting resources showed that healthy values in terms of SOC, SHIS and OBQ scores were associated with better perceived well-being. To report higher SOC were related to moderate-intensity physical activity and better OBQ was related to high-intensity exercise. All three health resources were related to a lack of sleeping problems. These results support the importance of maintaining sustainable health-promoting strategies during higher education, as has been described in previous research [17, 26].

### Health and health-promoting resources

In the results, most students reported good general health and good general well-being. This is consistent with previous research among nursing students [39]; however, previous studies have also found that general health and well-being decline during the 3-year bachelor programme [40]. Examples of health-promoting strategies that higher education can implement to maintain good general health and general well-being and create more incentives for a health-promoting perspective include integrating knowledge of sustainable working life into the programme through the courses’ content and pedagogical structure. This can be done during clinical placement and via reality-based cases where students train and learn to manage different scenarios, and reflect on their performance in these scenarios with the support of various reflection models. As another example, the students can develop a portfolio, in which they can track their progress during their programme and perform tasks with a focus on promoting a sustainable working life. Furthermore, health-promotion strategies may involve recruiting experienced students as Supplementary Instructors to support other students in their studies.

In this study, health-promoting resources were measured by the SOC, SHIS and OBQ scores. In earlier studies, SOC has been found to be strongly related to health [41], and it has been suggested that the SHIS and OBQ are reliable salutogenic health measurement instruments [21, 35]. The mean SOC score was 59.09 in the current study, which is somewhat lower than that reported in a previous study based on a general Swedish population aged 20 and 30 (63.3 and 70.0, respectively) [42]. The differences may be related to the different contexts that are, aspects related to the student situation and health. A health-promoting strategy could involve finding interventions to strengthen the salutogenic dimensions included in these three resources. Another health-promoting strategy could involve adding health-promoting lifestyles to the schedule of some programmes. Given that the Student Health Centres have knowledge of the students’ well-being, in cooperation with the student union, they could work proactively to prevent health problems and strengthen the study environment.

### Healthy lifestyles

In the current study, one-third of the participants met the recommendations of physical activity of at least 150 min of moderate-intensity physical activity per week, and nearly half of the participants engaged in at least 60–90 min of high-intensity exercise per week. This is in line with the general population in Sweden [43]. Previous research has stated that higher education students’ health-related lifestyle give cause for concern and suggests that universities need to focus on health-promotion work among students [44]. However, it is lower than previous research among nursing students, in which three-quarters met the recommendations for physical activity in their first year of higher education [39] although this rate declined during the 3-year bachelor programme [40]. Based on research from other industrialised countries, around two-thirds met the recommendations for physical activity of 150 min of moderate activity per week, in Australia and Spain [6, 45], whereas only one-third did so in England, Greece and the United States [4, 7, 44]. The differences between countries in terms of students’ physical activity may be due to variation in social, socio-economic and cultural factors. They may also be due to a phenomenon reported in Gibson et al.’s (2016) study, where students experience a major change at the beginning of their university studies; as a result of this change, they have to adjust to a new environment and choose healthy behaviours, including physical activity, based on their own motivation [46].

In our study, sex was not associated with the level of physical activity, but other research has shown that sex is associated with physical activity, with women having almost twice the risk of insufficient physical activity compared with men [7, 44]. Regarding the difference between men’s and women’s physical activity levels, there may be various reasons for this disparity. For example, given that women usually have the greatest responsibility for the family and household [47], they may have less time to spend on leisure activities. This can impact their chances of attaining balance in life. The sex difference can also be due to different categorizations and measurement methods, meaning that it may be a result of differences in the way the research studies were conducted.

Most students had no sleeping problems; however, there were variations between the students in the different programmes. Nevertheless, 4 out of 10 students reported sleep problems. This is in contrast to previous research among nursing students, where only 1 in 10 students reported sleep problems during the first year of higher education [39] although sleep problems did increase during the third year [40]. Sleep could have an impact on anxiety and depression [13], and is essential to serve several important physical functions, such as recuperation from infectious diseases and consolidation of memories [48]. A recent literature review and meta-analysis showed that sleep disruption is high among medical students, and this severely impairs learning ability and affects academic performance [49]. One potential reason for difficulty sleeping is anxiety and stress about the person’s economic situation. Students usually have limited financial resources during their studies. Another reason may be performance anxiety or that their results fell short of their expectations. The increase in sleep problems at the end of the study period may be due to concerns about not having enough knowledge to cope with the upcoming professional role. Thus, studies aiming to follow up on sleep habits during their studies, and interventions to improve sleep quality for students within healthcare and social work, are recommended.

The results show that the daily intake of vegetables among the students is in line with studies among students in the United States [7]. Of all students, most were non-smokers and non-daily snuff users; over half the students reported low alcohol consumption. The prevalence of smoking and alcohol consumption among the participating students is in line with research from other countries in Europe [40, 44, 45].

### Associations between general health, health-promoting resources and lifestyle factors

This study shows an association between self-reported good general health and higher SOC, SHIS and OBQ scores. Self-reported good general health and high SHIS and OBQ scores were associated with better perceived well-being, performing high-intensity exercise and being less sedentary. Being less sedentary and performing moderate-intensity physical activities were also associated with higher SOC scores. This is confirmed by previous research that found associations between perceived health and the degree of physical activity. Being intensively physically active entails a better perceived health-related quality of life [50].

The current study also shows that having no sleeping problems was significantly associated with self-reported general better health and higher SOC, SHIS and OBQ scores. A national survey in Norway including all higher education students showed that the mean sleep duration on weekdays was just under 7.5 h per night and did not meet the students’ self-reported sleep needs or sleep recommendations; in contrast, on weekends, the mean sleep duration was almost 8.5 h per night. In the last decade, the proportion of higher education students reporting sleep problems has increased, rising from 23% in 2010 to 31% in 2018; moreover, 22% of men and 34% of women met the criteria for insomnia [51]. Sleep problems are associated with poorer academic skills and results among higher education students [52, 53]. Since sleep problems are both prevalent and increasing [51] and influence academic results [52–54], sleep interventions can be important in health promotion work to improve health outcomes and overall academic performance among students [54].

This study shows associations between a daily intake of vegetables and self-reported good general health, combined with higher SOC and SHIS scores. Based on previous research, it appears to be common among students in higher education to have an unbalanced diet; for example, they may skip breakfast regularly or not reach the general vegetable intake recommendations [4, 6, 44]; moreover, they may experience unfavourable and differential changes to their dietary intakes and diet quality during the transition to university life [55]. Higher education students’ health behaviours seem to be risk factors that need attention, and N Yahia, D Wang, M Rapley and R Dey [7] argue that students in the United States, especially male students, might benefit from nutritional training programmes that focus on translating theoretical nutritional knowledge into daily-life applications, whereas female students would benefit from reducing the time spent in sedentary activities and being more physically active [7].

Higher education has an important part of student life. Therefore, higher education institutions must increase their accountability and willingness to design health-promotion interventions in the teaching and learning environment, focusing on multiple lifestyle issues [45]. Moreover, they should include health promotion in their core values [44] to prepare the students for future sustainable working life. In this health-promotional work, higher education institutions can implement the portfolio as an educational method; this can increase students’ self-efficacy, as well as forming the basis for self-evaluation and providing tools for lifelong learning [56, 57].

An unexpected result in the current study was that most students reported good overall health and good overall well-being, although 4 out of 10 students also reported sleep problems. The cause of this relationship is unknown, and this result needs to be further investigated. Qualitative studies with either focus group interviews or individual in-depth interviews could provide a deeper understanding of the three health resources related to sleep. This cross-sectional study provides ideas for further research into factors that affect students’ health. It would be particularly interesting to follow up on how higher educational institutions can design health-promotion interventions in the teaching and learning environment, with a focus on lifestyle issues.

### Strengths and limitations

In aiming to improve the research standard and reduce publication bias, the study protocol for this study was previously peer-reviewed and published [32], which must be considered a strength. The primary limitation of the cross-sectional study design is that it cannot establish a cause-and-effect relationship. Another limitation of cross-sectional studies is that individuals are not followed up over time; however, this cross-sectional study represents a baseline, with follow-ups for coming longitudinal studies described in the study protocol. This study is also the first step in a data collection series that is, a baseline aiming to follow up students on higher educational programmes in the healthcare and social work fields and the post-graduation period as new professionals. Thus, this study provides an important overview of health, health-promoting resources and lifestyle factors reported by first-year students in higher education within these fields. To the best of our knowledge, there is sparse research that has targeted the healthcare and social work field to predict possible salutogenic factors rather than pathogenic factors. A strength of the current study is that validated instruments developed to catch health-promoting factors have been used. Since all the students were invited to participate, there was no selection bias in this study. Self-report bias can occur when the people who complete the questionnaire are the sort of people who like to complete questionnaires; this can influence and limit the results. However, some limitations must be considered. Power calculation is often a recommendation for calculating statistical analyses and for appropriate generalisation to the population. This study was considered a total population study representing all students at six university programmes in health care and social work. Thus, the results can only be generalised to this population.

## Conclusions

Most first-year Swedish students from six universities in higher education within healthcare and social work programmes seem to report good health, as well as important health-promoting resources reflecting high SOC, SHIS and OBQ scores and a healthy lifestyle. High-intensity exercise, no sleeping problems and non-smoking are positively associated with general health and health-promotive resources. Higher education is an important arena for health promotion in multisectoral health improvements, supported by a teaching and learning environment that focuses on lifestyle issues. This study contributes to delineating the characteristics of students in the healthcare and social work sectors.

## Data Availability

The datasets and materials used and/or analysed during the current study are available from the corresponding author upon reasonable request.

## Abbreviations

OBQ: Occupational Balance Questionnaire
SHIS: Salutogenic Health Indicator Scale
SOC: Sense of coherence
